# Provider-reported experiences, barriers, and perspectives on genetic testing as part of autism diagnosis

**DOI:** 10.1371/journal.pone.0296942

**Published:** 2024-02-05

**Authors:** Amy Wang, India D. Little, Dennis Carter, Stephanie Pham, Madeline Piper, Gabriela M. Ramírez-Renta, Sydney Telaak, Chris Gunter

**Affiliations:** 1 National Human Genome Research Institute, Social and Behavioral Research Branch, Bethesda, Maryland, United States of America; 2 National Institute of Mental Health, Office of the Clinical Director, Bethesda, Maryland, United States of America; 3 Johns Hopkins University and National Institutes of Health, Genetic Counseling Training Program, Baltimore, Maryland, United States of America; 4 National Human Genome Research Institute, Bethesda, Maryland, United States of America; Chinese Academy of Medical Sciences and Peking Union Medical College, CHINA

## Abstract

Several professional organizations recommend conducting genetic testing as part of the autism diagnosis process, as it can provide additional information and benefits for autistic people and their families. However, there is disagreement among autism communities about whether genetic testing reflects autistic people’s best interests. In practice, rates of clinical genetic testing for autism are much lower than diagnoses, creating a large gap between clinical guidelines and real clinical encounters. To investigate one potential source of this gap, we interviewed 14 healthcare providers about the autism diagnostic process and their actions related to autism genetic testing. We recruited a sample of primarily Ph.D. level-psychologists and analyzed our qualitative data using a five-step framework analysis method. Participants generally had positive or mixed views of genetic testing in autism. They described their current experiences of implementation of genetic testing, including that they did not often find it changed their clinical practice. Only some providers recommended it to everyone receiving an autism diagnosis. They also listed factors which discourage families from getting testing, including high costs, families feeling overwhelmed, other support needs taking priority, and ethical implications. Notably, providers highlighted a trend of referring patients to research genetic testing rather than clinical testing, which may provide a cheaper and easier alternative but is not likely to return results to participants. Finally, participants felt they needed more training in genetics and listed specific topics of uncertainty. Our research highlights a need to further educate clinicians in the uses and limitations of genetic testing for autism and suggests content areas of focus for genetics educators.

## Introduction

Although prevalence rates of autism are rising, autistic people still face many barriers towards getting an official diagnosis. Autism can be reliably diagnosed by age two [1], but the median age at diagnosis occurs later at 4.1 years old [2], which indicates a need to improve access to diagnostic services.

Clinical genetic testing may be used as part of the autism diagnosis process, or after receiving a diagnosis as a source of additional information. The heritability of autism is estimated to be 64–91% [3], with hundreds of genetic variants so far found to be related [4]. Both the American College of Medical Genetics and Genomics [5] and the American Academy of Child and Adolescent Psychiatry [6] recommend conducting genetic testing at the time of an autism diagnosis. The most commonly used genetic testing option for autism has been chromosomal microarray (CMA) [7]. More recent data finds that exome sequencing outperforms CMA by improving diagnostic yield for cases with primarily autism to an average of 16%, and to an average of 37% in a more heterogeneous group of cases with intellectual disability and autism [8]. For this reason, exome sequencing is now the recommendation as a first-tier test for NDDs [8]. Among the minority of patients who received pathogenic findings on their CMA or Fragile X testing, one study found that 72.2% were given additional medical recommendations based on their genetic results, such as seizure monitoring or referrals to subspecialists [9]. The testing can also help parents of autistic children understand why their child is autistic and may also facilitate access to additional services, such as early intervention programs, and educational, disability, and employment services [10,11].

However, uptake for genetic testing remains low. Completion of both CMA and Fragile X testing is reported to be as low as 3% [12]. Much of this low uptake may result from healthcare providers (HCPs) not providing a genetic testing recommendation. In one study, 83% of parents reported that their doctors never provided information about genetic testing for autism, despite most parents expressing an interest in this topic [13]. HCPs may be less likely to discuss genetics due to low genetic literacy levels. Genetic literacy is defined as the sufficient understanding of genetics concepts to make decisions on personal well-being and genetics issues [14]. First year pathology students showed lower understanding of genetics concepts compared to non-genetics concepts [15], and medical students frequently self-reported having insufficient genetics knowledge [16]. Increasing HCPs’ engagement with genetics is crucial because parents who received genetic testing information from an HCP were 4.65 times more likely to get their child tested [17], and having a doctor’s recommendation is the most predictive factor in whether a patient completes genetic testing [18].

Many people from autistic communities also express concerns about genetic testing, and personal or ethical reasons may further contribute to low testing rates. In 2022, the Autistic Self Advocacy Network (ASAN) released a statement on their stance about genetic research and autism. They expressed several concerns about genetic research, with primary worry being that such research could be used to develop “cures” for autism [19], terminology which both ASAN and the authors of this paper do not endorse. Additionally, survey data from autistic adults reveal that 49% do not believe that genetic testing for autism should be conducted at all, and 40% believed that genetic testing was only harmful [20].

It remains unclear how aware HCPs are about the benefits and concerns towards genetic testing for autism. The current study conducted qualitative interviewing of HCPs who were involved in the diagnostic process for autism, primarily focusing on providers who are licensed PhD-level psychologists and therefore currently do not order genetic testing in the US themselves (as opposed to MDs). In our sample the PhD providers, largely psychologists, spend significant amounts of time with the family to provide an autism diagnosis, and make the decision on whether or not to refer the families to another HCP who can order clinical genetic tests. Our first aim was to assess HCPs’ thoughts about the usefulness of genetic testing, and how they currently use genetics in their clinical practice. A 2023 study [21] reported that although 87% of a sample of child and adolescent psychiatrists–whom in the US would be MDs and can order genetic testing–“agreed that it is their role to discuss genetic information” with families, a full 36% of those currently ordering genetic testing rated their own knowledge of genetic testing practice guidelines in psychiatry as poor or very poor. To illuminate gaps for HCPs who feel they could benefit from additional education, our second aim was to ask our participants what they wished to learn more about regarding genetic testing for autism. These responses can help inform strategies to increase HCPs’ awareness of genetic testing and improve their confidence in discussing genetics with their patients.

## Materials and methods

The National Human Genome Research Institute’s Institutional Review Board (IRB) reviewed the research protocol and ruled it exempt (protocol 000670).

### Participants

14 HCPs participated in the study. Participants were eligible if they were 18 years old or older, could speak and read in English, and were involved in the autism diagnosis process within the past six months. From February 2022 to July 2022, we recruited participants using snowball sampling, as described in Parker et al [22]. CG and IL generated an initial recruitment list based on their existing contacts in the field, primarily focusing on PhD-holding psychologists. Early participants then recommended colleagues or posted the invitation to a relevant group email list. We attempted to contact HCPs who were diverse in race, gender, and geographic location in the United States, first emailing potential participants and then screening for eligibility through a phone call. Self-reported demographic information of the participant pool is shown in Table 1; more specific data on socioeconomic status was not recorded. The final sample group was mostly Ph.D. level psychologists, and all of them saw primarily pediatric patients. Participants provided oral consent before beginning the interview, as approved by IRB, and noted in the transcripts [see S1 File for exact consent language]. Recruitment ended once the team determined that we reached thematic saturation.

**Table 1:** Participant demographics (N = 14).

Age, M (SD)	45.8 (9.4) years
Race, N (%)	12 (85.7%) White, non-Hispanic 1 (7.1%) White, Hispanic 1 (7.1%) Multiple races (Black and White)
Gender, N (%)	12 (85.7%) Female 2 (14.3%) Male
Occupation, N (%)	12 (85.7%) Psychologist 1 (7.1%) Psychiatrist 1 (7.1%) Pediatrician

### Interview structure

Four research assistants (DC, IL, SP, ST) conducted semi-structured interviews, which typically lasted 30 minutes to one hour. The interview consisted of questions broadly related to their experience with diagnosing autism, opinions about support offered for autism, and awareness of genetic testing (see S1 for interview guide). We conducted interviews using Microsoft Teams, and the audio was transcribed and stripped of identifying information by National Capitol Contracting. Subjects were compensated with a $50 gift card for their time.

### Data analysis

We conducted the qualitative data analysis using framework analysis as described by Ritchie & Spencer in 1994. The process includes five steps: 1) familiarization, 2) identifying a thematic framework, 3) indexing, 4) charting, and 5) mapping and interpretation. The familiarization phase involves studying transcripts to note key ideas and recurring themes. The second phase, identifying a thematic framework, involves using the key concepts identified during familiarization to develop a codebook, which is used to classify the data (see S2 File for codebook). Indexing involves identifying portions of the transcripts which correspond to a particular theme in the codebook. Our team used a combination of Microsoft Excel and NVivo for this process. Charting involves removing pieces of data from their original transcripts and placing them in charts based on the codebook’s themes. We used Microsoft Word to chart our data. Finally, mapping and interpretation involves analyzing key characteristics from the charts to guide interpretation of the dataset [23]. Steps 1, 2, 4, and 5 were primarily led by AW, and step 3 (indexing) was completed by four independent coders (DC, CG, GRR, AW). Coders met to discuss results and interpretations, and the codebook themes were adjusted as the analysis process progressed.

### Community involvement

Our research team includes autistic coauthors, who led the study’s design and conceptualization, and offered insights in interpretation of findings and manuscript editing.

### Language choices

In this manuscript, we use the term “autism” when describing the condition and the term “autistic” when describing individuals. We use identity-first language to reflect the preferences of many autistic people [24,25], though we recognize that preferences may differ among individuals. This manuscript may use different language choices (such as “ASD” or “autism spectrum disorder”) when using a direct quote from a participant.

## Results

Participants discussed a variety of topics broadly related to the diagnostic process for autism. In this section, we summarized participants’ interview responses, which may not necessarily reflect the authors’ beliefs or the wider scientific literature.

### HCPs’ experiences with genetic testing referrals

Several participants expressed not receiving formal genetics training, with most knowledge being from on-the-job training only. Although many were aware that genetic testing was the standard of care, some revealed that they were not trained in how to order or interpret a genetic test. One psychologist reported:

“*I was never trained in ASD, so no. I went to a School Psychology program. … We maybe had, like, one hour on autism. … And so my training and especially in genetics of autism came on the job as part of research that we were doing*.”

(Participant 1, psychologist)

A psychiatrist participant reported similar gaps in their training:

“*People were starting to specifically say this [genetic testing] is standard of care. … But as a fellow, we didn’t always get much training on how to order it. … So, yes, hard to order, hard for me to interpret because I didn’t get any training in that*.”

(Participant 9, psychiatrist)

Many psychologist (PhD) participants reported being unable to directly make referrals for genetic testing, given the current US health framework. Instead, they instructed their patients to return to their pediatrician or primary care physician (MD) to request a genetic testing referral. Some participants also made sure to include genetic testing as a recommendation in the medical record to ensure that the physician will see it.

“*I can put in an order here for a child to go to a feeding clinic, or to go to a sleep clinic, or be seen in neurology, I can put in those referrals, but for some reason I can’t order labs as a psychologist, and genetics is considered a lab order*.”

(Participant 3, psychologist)

Participants varied in how often they recommended genetic testing, with some participants recommending it for every patient and some recommending only in specific cases. Even those who reported recommendation to every patient said they gave it with varying levels of urgency, depending on the specific characteristics of the patients. These characteristics include intellectual disability, global developmental delay, dysmorphic facial features, family history, or seizures.

“*We do often make recommendations for genetic testing. There are some kind of profiles of children where I am more apt to do that, like, you know, for some families, I strongly recommend you do this right now. For others, it’s this is something you might consider doing at some point*.”

(Participant 11, psychologist)

### Perceived utility of genetic testing

All participants expressed some positive benefits of genetic testing, although benefits may vary depending on whether the test yields a positive finding.

One of the most commonly stated benefits was the ability for genetic testing to inform medical trajectory. In particular, genetic information can predict symptoms and co-occurring conditions to monitor for in the future. This can help manage parents’ expectations about the course of their child’s condition. One participant said that knowing a specific genetic variant could help parents connect with other families who have experience with the same variant.

“*Actually sometimes they’ll ask them to connect to other families who have a child with the same condition. You know, there’s dozens if not hundreds of Facebook groups now for children with all these different genetic variants. And parents really, I think, get a lot from that*.”

(Participant 10, pediatrician)

Another commonly reported benefit was the ability for genetic testing to guide family decision making. If a variant related to autism is identified, further genetic testing in the family can suggest whether the variant is de novo or inherited. It may also offer the chance for other family members to also get genetic testing to better understand themselves.

Less commonly cited benefits included helping families better understand autism, gaining a fuller clinical picture, and enabling future scientific developments. Discussing genetics can help parents better understand the causes of autism, particularly the biological basis of the condition. The information can also help people believe in the autism diagnosis more. As one participant described:

“*What I’ve found just in the real world out there, having a genetic result is more believable to people than autism, which seems to be an opinion. So like a school or someone is like, ‘Oh, that’s something I can hang my hat on. They have a 16q duplication or deletion,’ as opposed to, ‘They have autism.’ You know, it’s a much more real thing for people to think about*.”

(Participant 6, psychologist)

More generally, a few participants said that genetic testing can be helpful to provide a more complete clinical picture, regardless of if the results are positive or actionable. The genetic test can rule out alternative medical explanations or may be useful simply for the sake of knowing. Additionally, several participants expressed that genetic testing could help advance future scientific developments. When more people get genetic testing, they felt, scientists may identify new genetic variants and develop personalized medicine.

However, roughly half of participants gave mixed opinions on genetic testing, saying that it frequently was not useful for them. In their experiences, tests often returned negative (no reportable genetic findings) or with a variant of unknown significance (VUS). Even when the test did return a positive finding, many variants lacked sufficient research to understand their clinical implications. For these reasons, genetic testing often did not change a participant’s clinical practice or medical recommendations.

“*At this point, I don’t know if they’re giving them anything else, you know, a whole lot of additional information or services. So, in the immediate, I don’t know if it’s that useful, but I think we really need to learn about this. And that we’re, I think, probably getting closer and closer*.”

(Participant 12, psychologist)

A few participants also expressed caution towards the ethical implications of genetic testing. In particular, they discussed the potential for genetic testing to be used for eugenic purposes, such as reducing the occurrence of autism.

“*Yeah, many of our centers have been accused of, what’s the word for it, not euthanasia, eugenics. Like if we’re able to do genetic testing in utero and see that your child has autism, what does that mean, you know? Does that mean you as a pregnant mother can do something about it? And if so, what is–if your identity is autism, what does that mean, you know? So there’s all kinds of really, really difficult questions that we’ll have to struggle with as we move forward*.”

(Participant 6, psychologist)

Uses of genetic testing and illustrative quotes are summarized in Table 2.

**Table 2:** Uses of genetic testing.

Use	Illustrative quote
Advance scientific discoveries	“I feel like the more information and data we have from families who have individuals on the spectrum, the more we’re going to identify different variants that do give us more information and guidelines.” (Participant 11, psychologist)
Gain fuller clinical picture	“I guess if my kid, you know, had an, an identifiable genetic disorder, I would want to know even if the treatment course didn’t change … even if it’s a, like a de novo mutation and doesn’t change my decision to have kids or like the heritability is really low or whatever, I think I would just still want to know to get a full clinical picture.” (Participant 13, psychologist)
Guide family decision making	“Well, I think if there are multiple family members with issues, or they want to continue to have children, certainly they would, that can be helpful for family planning … Or if they’re lacking information, if this is a child who’s, you know, in foster care or adopted, if they can get some more information from that.” (Participant 12, psychologist)
Help families understand diagnosis	“I think most importantly it helps parents better understand the medical roots, the neurologic roots, the genetic roots of their child’s developmental disorder; that it’s not just a matter of the child’s not learning to do things; it’s not a mental health condition per se; it’s really biologically rooted. And I think that makes it quite clear, especially if the test is positive.” (Participant 10, pediatrician)
Inform trajectory and medical recommendations	“If we know the genetic disorder, we also know the concomitant other types of diagnoses that might come along with it, like language disorders, GI problems, anxiety. You know, whatever it might be, different genetic disorders have different other symptoms that we know go along with them that will help us understand that child better.” (Participant 6, psychologist)

### Factors contributing to low genetic testing uptake

Participants listed several factors contributing to families not following through with genetic testing recommendations. The most commonly cited reasons were insurance and cost barriers: the high cost of genetic testing can prevent some families from affording other essential services for their child. As a solution to these costs, some participants recommended their patients to receive genetic testing through research studies. Participation in research can help patients get genetic testing for free and even receive compensation, avoid wait times, and sometimes do a larger gene panel, although the return of results may be slower or non-existent. Likely due to our sampling method, the research project most frequently mentioned was Simons Powering Autism Research for Knowledge or SPARK (www.sparkforautism.org).

“*So now I feel blessed, because I can say, oh I hear that it’s going to cost you a lot of money, do you instead want to participate in SPARK? It’s going to take longer to get the results, they’re going to do a probably a bigger panel in some ways … And they’ll keep your DNA so if there’s more findings they’re be able to double check on that in the future, so you’ll always be able to get results*.”

(Participant 3, psychologist).

One participant also described parental concerns about discrimination based on genetic status. Although the Genetic Information Nondiscrimination Act (GINA) protects against genetic discrimination, the participant explained how this law was not well-known among both parents and providers:

“*There are laws to protect people now against that kind of discrimination. If you don’t know, you should look up GINA. … Not many people [know about it], including professionals in my own field. … And with the Affordable Care Act, no child or adult could be denied services based upon a preexisting condition. It wasn’t always that way, but families still don’t know or understand that. But it does impact other types of insurance where there aren’t those protections–life insurance, disability insurance, and so on. So some families would rather not have that information if a child can be discriminated against based upon that, those genetic tests*.”

(Participant 10, pediatrician)

Another common factor was families feeling overwhelmed at the time of diagnosis. One participant described the process as being highly emotional for families, which causes HCPs to become hesitant to share additional genetic recommendations.

“*I think sometimes when something’s hard, people are less inclined to bring it up, especially if it’s already hard to share an initial diagnosis. And I feel that too, like, wow, they’re already struggling with the diagnosis and now I’m layering on, ‘oh, and it could be genetic.’ And, you know, they wanted to have more kids*.”

(Participant 1, psychologist)

One contributor to these overwhelming feelings is the many medical recommendations that families receive at the time of a diagnosis. Many are unable to follow up on all appointments, causing the need for families to prioritize getting therapeutic services for their child over seeking genetic testing.

“*I think also because we’re serving a demographic that has a lot less resources for the most part, a lot of the questions are a lot more around, how do I prioritize what I need to do? And if I’m thinking about that, genetics is certainly important, but I try and find things that I know parents are going to be able to prioritize, even just another doctor’s appointment can be really rough because it means taking time off of work to be able to get to the doctor’s appointment to be able to do that*.”

(Participant 3, psychologist)

Other commonly reported factors were families not believing that genetic testing would benefit them (particularly among parents who were not planning to have additional children) and long waitlists for genetics clinics. These genetics clinics often have even longer waitlists than those seen for autism diagnosis, and wait times for genetic counseling can pose additional challenges:

“*There’s so many times you hear the story that positive genetic findings and they just got a letter with the lab results and they didn’t know what that meant, and then, to see a genetic counselor is like a nine month waiting list or something. So families are sitting with this genetic finding, and then they’re just Googling, and we know the dangers of that*.”

(Participant 3, psychologist)

Less commonly cited reasons were parents feeling intimidated by genetic testing, low genetic literacy, the procedure being too difficult for the child (particularly difficulties surrounding the blood draw), cultural differences, and travel barriers. A summary of contributing factors and illustrative quotes are presented in Table 3.

**Table 3:** Factors contributing to low genetic testing uptake.

Contributing factor	Illustrative quote
Cultural differences	“I’m sure there’s religious and cultural things too that play a role. You know, if you believe that this was god’s will, maybe you’re not, you don’t care so much about genetics.” (Participant 1, psychologist)
Feeling intimidated by genetic testing / afraid to know	“They might be a little bit scared of it because it seems so concrete then, right? It’s like, depending on how much in denial they are about the diagnosis, having something real like a genetic disorder makes it–it just shifts the whole narrative in terms of how they would perceive their child, right?” (Participant 6, psychologist).
Insurance	“Talking about insurance barriers, there’s been so many times where we put that referral in, and the parents come back and say, my insurance won’t cover it, or, you made that referral, did you know it was going to cost me $10,000?… And I have a hard time when parents are telling me it’s going to cost them the ability to provide interventions for their child, versus going to get testing.” (Participant 3, psychologist)
Low genetic literacy	“The most I get about genetics is, ‘Is autism genetic?’ Like that’s the level that we’re working at. And so I never get asked about genetic testing, like directly, which is telling, I think, of how many or how few people think about it.” (Participant 13, psychologist)
Overwhelmed or not a priority	“Some families, the information is just overwhelming. And they just want their child to get help right now for what their needs are right now and so they’ll focus on the interventions and the therapies and not really worry or think about the medical aspects of it.” (Participant 10, pediatrician)
Perceived as not having benefits	“For the families it’s like, ‘Well, who cares? I know, I don’t need to do genetic tests, it’s not going to tell me anything, it’s not going to add anything, so you get more stress.’ So I think it’s more of that type of–and the more we normalize the genetic testing, then it becomes easier for families to, I think, accept it.” (Participant 6, psychologist)
Procedure difficult for child	“No child ever is, you know, or no parent is ever excited to have their child have a blood draw. … just that kind of trauma that’s associated with that sometimes it’s not worth it to families.” (Participant 5, psychologist)
Travel needed for appointment	“If it’s another appointment, they have to travel somewhere to kind of go get a blood draw. Is that close to them? Do they have to take a day of work to go get it done? So, I’m sure there’s a lot of logistical barriers, transportation barriers that probably also get in the way of that not getting collected if it can’t get collected that same day of the appointment.” (Participant 7, psychologist)
Wait times for genetics clinics	“We have a little bit of a waitlist for genetic testing. So, I would say families on average, if they follow through with the appointment, get it three to six months after their formal diagnostic evaluation.” (Participant 2, psychologist)

### Topics that HCPs wish to know about genetic testing

Aiming to identify areas in which future education could be targeted, we asked participants which topics in genetics they would like to learn more about. Several participants expressed that they only wanted to know about topics that were relevant to their clinical practice. Specific topic areas that emerged fell into four categories: utility of genetic testing, understanding of genetics, how genetic testing works, and how to discuss genetic testing with patients. Participant responses were paraphrased and summarized in Table 4.

**Table 4:** Genetics topics healthcare providers wish to learn about.

Utility of genetic testing	Why recommend genetic testing? What can the test tell you, and what can’t it? How does it impact the family’s day-to-day life? How does it impact the clinician’s work?
Understanding of genetics	What are the trajectories associated with different genetic syndromes? What is the difference between variation among humans in general vs. when it is a concern? What is methylation?
How genetic testing works	What is looked at in genetic testing? What kind of genetic test should I refer my patient for? How do you interpret the results? What is the yield of genetic testing? What is the precision of genetic testing? How does a microarray work? What does the family need to do for it? What is the difference between no variant vs. known variant vs. VUS? Will a VUS ever have known significance? How do you check if a VUS had been updated? How often do you redo the genetic testing?
How to discuss genetics with families	What are plain language resources that can be shared with families? How much of the results do you report to the family? What kind of training do genetic counselors have with autism? How does a genetic counselor talk to patients? What are families’ experiences with genetic testing?

## Discussion

Our work provides a perspective from psychologists and physicians who diagnose autism to assess their experiences with genetics. Although genetic testing has been proposed to help facilitate the autism diagnosis process [5,6], the providers we interviewed were clear that it would generally be recommended after an autism diagnosis, and not before. It should be noted that our interviewees were primarily Ph.D. psychologists and not M.D.s, and therefore in the current American system they are largely unable to make direct referrals for genetic testing on their own.

Participants varied in their attitudes towards genetic testing, which echoes the variable responses collected in Barton et al [26]. These interviews did suggest some situations where genetic testing may aid in the autism diagnostic process. Genetic testing can help families better understand the causes of autism and may alter people’s perceptions about the condition, for better or for worse. Genetic explanations have been proposed as a possible method to reduce stigma surrounding mental health, although evidence is mixed [27–29]. Greater efforts should be made to reduce families’ stigmatic attitudes towards mental health diagnoses, and reducing stigma may help families feel more willing to seek diagnostic services for their children.

However, genetic testing may also add more difficulties to the diagnostic process. Families already undergo long wait times for diagnostic services, and genetics services often add even longer wait times. High costs for genetic testing can put financial strain on families, which may impede their ability to pay for other autism services. Furthermore, families were often reported as being overwhelmed by the diagnostic process, and the additional appointments can contribute to their stress.

Responses were mixed regarding the ability for genetic testing to affect HCPs’ clinical practice. Many participants expressed that they often lacked the training to interpret the findings by themselves, and ultimately the test results did not change any clinical recommendations related to autism or other conditions. The providers understood many families chose not to pursue clinical genetic testing because they believed it would not benefit them. In fact, a few participants directed their patients to prioritize other services over getting genetic testing.

Participants also discussed genetic testing as a guide for family planning. However, multiple problems exist with this planned usage: first, autism has an extremely heterogenous genetic profile, and genomic technologies are of limited and/or questionable use for that clinical application. Second, members of the autistic community and the genetics community do not always agree on where the line between family planning and eugenics should be drawn [30]. Autistic self-advocates raise many legitimate concerns about genetic research, including examples of historical misuse of genetics [19]. We want to clearly state that autistic identities are valued, and any eugenic applications should be abhorrent to biomedical researchers and clinicians. We suggest that an understanding of the facts and limitations of genetic research, along with true participatory engagement, is crucial to ensure any partnership with autistic individuals and their families.

One unexpected theme from our analysis was the replacement of clinical genetic testing referrals for research projects in genetics, specifically the SPARK study [31], which was also noted briefly in Abreu et al [32]. Multiple providers stated that they refer families to SPARK for “genetic testing” regularly because it’s “easier” (saliva is taken instead of blood and this can be done at home), it does not cost the family, and the project will revisit sequencing results as more genomic information associated with autism is learned over time. Unfortunately, this research testing cannot be a true replacement for clinical genetic testing, as clinical results will rarely be returned to participants. As the FAQs on the SPARK website state [33]:

“*Will everybody in SPARK receive genetic results? No. Because SPARK is a research study, our genetic analysis is not like a clinical genetic test or commercial sequencing service. SPARK provides genetic results in the form of a clinical report only if we discover a genetic change associated with autism*.”

We note that SPARK is so large, with over 100,000 individuals with autism and 175,000 family members participating (as of January 2023), that our data suggest it will have a measurable effect on the uptake of clinical genetic testing in the United States through the decisions of providers such as those in our study to refer families there rather than to formal clinical testing. This effect might not have been seen in previous studies looking at MDs who can order clinical genetic testing, as opposed to our sample of PhDs who make recommendation decisions but can refer to research testing. We suggest that future studies measuring clinical genetic testing for autism in the US need to take this variable into account.

Overall, our data show that HCPs are interested in learning about genetics, but lack the training needed to fully understand testing results and discuss genetics with their patients. Increasing HCPs’ genetic literacy will enable them to understand the nuances of genetic testing and help patients reach informed decisions about their health. To our knowledge, this study is the first to identify topic areas in genetics that HCPs wish to learn more about, which can inform content for genetics education. Specifically, several participants expressed only wanting to learn information that would be useful to their clinical practice, and the topic areas they discussed likely reflect the concepts that would be most useful for them. Educational interventions should emphasize the utility of genetic testing, how genetic testing works, and methods to discuss genetics with patients.

There were several limitations to this study. First, all of our participants worked in pediatric clinics. Adults seeking autism diagnoses experience unique challenges which were absent from our responses. Additionally, most participants expressed that although they largely could not order genetic tests themselves, they recommended genetic testing to their patients, which contrasts with literature showing that most HCPs do not offer genetic recommendations [17,34]. This finding may be due to participation bias, with more participants who are interested in genetics choosing to participate in a research study asking about genetic testing. Additionally, given our method of snowball sampling, most of our participants worked in academic settings, which may make them more aware of current research in autism and more likely to be affiliated with large research projects such as SPARK. It is also possible that the frequency of genetic recommendations is underreported in the literature: while many studies quantify this rate using data reported from families [13], our HCP participants stated that they often make recommendations to their patients that patients later forgot they had discussed. Finally, our study investigated one heterogeneous group’s perspective about genetic testing for autism and should be considered along with experiences of autistic individuals or families to fashion a more complete understanding of autism diagnoses.

In addition to their perspectives on the diagnostic and testing processes, the providers interviewed here expressed the need for further educational interventions. As shown in Table 4, they specifically identified topic areas of concern or interest, suggesting immediate next steps for genetics education which could help inform providers working in this area.

## Supporting information

S1 File Interview guide. (DOCX)

S2 File Qualitative codebook. (XLSX)
