# Supplementary material for: Provider-reported experiences, barriers, and perspectives on genetic testing as part of autism diagnosis
Source: PLoS One. 2024 Feb 5;19(2):e0296942. doi: 10.1371/journal.pone.0296942 (PMC10843127; doi:10.1371/journal.pone.0296942)
Supplement: S1 File — (DOCX) [file pone.0296942.s001.docx]

Thank you for agreeing to participate in this research project conducted by the National Human Genome Research Institute. The goal of the project is to learn more about your experiences surrounding diagnosing autism spectrum disorder, or ASD, and the use of genetic testing for ASD. We are going to ask you a few questions; please respond however you would like. There are no right or wrong answers and you may share as little or as much as you would like.

All your responses are confidential and will be de-identified.

This call will also be recorded, and the recording will be used to document your responses. Once it has been transcribed, the recording will be deleted. Is that alright with you?

Do you have any additional questions for me?

**START RECORDING CALL**

By continuing with this interview you are confirming that you have read the consent form and freely and voluntarily consent to participate in this research study. Is that correct?

First, I’d like to confirm a few demographic questions with you. Would that be alright?

Demographic/Background Questions:

1. How would you describe your race or ethnicity? How about gender?
2. Could you tell me about your current occupation?

Interview Guide:

1. Experience with/Role in diagnosing ASD
   1. **Now I am going to ask some questions about your experience in the diagnosis process for ASD.**
   2. How would you describe your role in the diagnosis process?
   3. At what point in the diagnosis process are you involved?
   4. In your experience, what are the barriers to assessing and providing autism diagnoses?
2. Thoughts and Opinions on Support Offered
   1. **Could you tell me more about an individual or families’ needs at this point in the ASD diagnosis?**
   2. How helpful do you think the kinds of support offered to families or individuals are?
   3. In your experience, what resources can those diagnosing ASD provide to the families or individuals?
   4. Can you describe your experience with offering referrals or resources at this point in the process?
3. Awareness of Genetic Testing for ASD
   1. **Could you describe your experience with genetic testing referrals for those ASD?**
      1. If offered, in your experience, when do families get the referral?
   2. From your perspective, how useful is genetic testing for someone with an ASD diagnosis?
   3. In your experience, how do families decide whether they will seek genetic testing?
   4. What, if anything, would you like to know about genetic testing for ASD?
4. **Is there anything about your experiences or beliefs that I didn’t ask you that you’d like to share?**
   1. Briefly summarize notes/main takeaways and ask for corrections/clarifications.

That concludes our interview. Thank you so much for your participation, your responses will be very helpful in our research. Do you have any questions for me?

**STOP RECORDING**
